# Immunogenicity of a chimeric *Plasmodium falciparum* merozoite surface protein vaccine in *Aotus* monkeys

**DOI:** 10.1186/s12936-016-1226-5

**Published:** 2016-03-15

**Authors:** James M. Burns, Kazutoyo Miura, JoAnn Sullivan, Carole A. Long, John W. Barnwell

**Affiliations:** Center for Molecular Parasitology, Department of Microbiology and Immunology, Drexel University College of Medicine, 2900 Queen Lane, Philadelphia, PA 19129 USA; Malaria Immunology Section, Laboratory of Malaria and Vector Research, National Institute of Allergy and Infectious Diseases, National Institutes of Health, Rockville, MD 20852 USA; Malaria Branch, Division of Parasitic Diseases and Malaria, Centers for Disease Control and Prevention, Atlanta, GA 30329 USA

**Keywords:** Blood-stage malaria vaccine, *Aotus* monkeys, Vaccine carrier protein

## Abstract

**Background:**

The production of properly folded, recombinant sub-unit *Plasmodium falciparum* malaria vaccine candidates in sufficient quantities is often a challenge. Success in vaccine immunogenicity studies in small animal models does not always predict immunogenicity in non-human primates and/or human subjects. The aim of this study was to assess the immunogenicity of a chimeric blood-stage malaria vaccine in *Aotus* monkeys. This vaccine candidate includes the neutralizing B cell epitopes of *P. falciparum* merozoite surface protein 1 (r*Pf*MSP1_19_) genetically linked to a highly immunogenic, well-conserved *P. falciparum* merozoite surface protein 8 (r*Pf*MSP8 (ΔAsn/Asp)) partner.

**Methods:**

*Aotus nancymaae* monkeys were immunized with purified r*Pf*MSP1/8 or r*Pf*MSP8 (ΔAsn/Asp) formulated with Montanide ISA 720 as adjuvant, or with adjuvant alone. Antibody responses to MSP1_19_ and MSP8 domains were measured by ELISA following primary, secondary and tertiary immunizations. The functionality of vaccine-induced antibodies was assessed in a standard *P. falciparum* blood-stage in vitro growth inhibition assay. Non-parametric tests with corrections for multiple comparisons when appropriate were used to determine the significance of differences in antigen-specific IgG titres and in parasite growth inhibition.

**Results:**

The chimeric r*Pf*MSP1/8 vaccine was shown to be well tolerated and highly immunogenic with boost-able antibody responses elicited to both *Pf*MSP8 and *Pf*MSP1_19_ domains. Elicited antibodies were highly cross-reactive between FVO and 3D7 alleles of *Pf*MSP1_19_ and potently inhibited the in vitro growth of *P. falciparum* blood-stage parasites.

**Conclusions:**

Similar to previous results with inbred and outbred mice and with rabbits, the *Pf*MSP1/8 vaccine was shown to be highly effective in eliciting *P. falciparum* growth inhibitory antibodies upon immunization of non-human primates. The data support the further assessment of *Pf*MSP1/8 as a component of a multivalent vaccine for use in human subjects. As important, the data indicate that r*Pf*MSP8 (ΔAsn/Asp) can be used as a malaria specific carrier protein to: (1) drive production of antibody responses to neutralizing B cell epitopes of heterologous vaccine candidates and (2) facilitate production of properly folded, recombinant *P. falciparum* subunit vaccines in high yield.

## Background

There have been measurable gains in reducing the global burden of malaria through the integration of control programmes involving insecticide-treated bed nets, indoor residual spraying of pesticides, and intermittent preventive therapy [1]. Nevertheless, the malaria burden remains high, with the World Health Organization reporting 198 million cases and an estimated 367,000–755,000 deaths worldwide in 2013. It is expected that the addition of an effective malaria vaccine to the battery of malaria control strategies would accelerate the decline in disease and promote long-term sustainable control. RTS,S, the first malaria vaccine to reach phase III clinical trials, is a pre-erythrocytic-stage vaccine based on the circumsporozoite protein of *Plasmodium falciparum* [2]. Initial reports suggest that vaccine efficacy may only be around 30 % in the most vulnerable target population of infants [3], with a higher efficacy of approximately 50 % in young children [4], but with concerns regarding the durability of protection. The efficacy of other malaria vaccine candidates that have been evaluated in phase II trials has not been impressive [5–10].

There is mounting agreement in the field that an effective malaria vaccine will require induction of immune responses to multiple, distinct target antigens. This concept is central in the development of whole parasite-based vaccines, including radiation (PfSPZ) [11, 12] and genetically (PfGAS) [12–14] attenuated sporozoite vaccines, infection-treatment pre-erythrocytic-stage vaccines [15, 16], and chemically inactivated whole blood-stage vaccines [17, 18]. However, these whole parasite approaches face significant challenges related to production, formulation, standardization, delivery, and safety that are less problematic for sub-unit-based vaccines. Other significant challenges associated with sub-unit malaria vaccine development have been encountered. These include difficulties in producing properly folded candidate antigens, polymorphism in T and B cell epitopes, and poor immunogenicity. Several years ago while working in the *Plasmodiumyoelii* model, the additional problem of antigenic competition was encountered when combining just two blood-stage vaccine components, merozoite surface protein 1 (MSP1_42_) and MSP8 [19]. This problem has also impeded the development of other multi-antigen malaria vaccine formulations [20–24].

For sub-unit malaria vaccines, a well-established strategy to enhance the immunogenicity of neutralizing B cell epitopes was adopted, namely the use of a highly immunogenic carrier protein. Taking advantage of the immunogenicity of MSP8, a chimeric protein with the conformational, protective B cell epitopes of MSP1_19_, fused to MSP8 was generated. Immunization with the chimeric r*Py*MSP1/8 vaccine induced high and comparable antibody responses against both *Py*MSP1_19_ and *Py*MSP8, resulting in nearly complete protection against lethal *P. yoelii* 17XL malaria [19]. The enhanced efficacy of the r*Py*MSP1/8 vaccine, in comparison to a combined formulation of r*Py*MSP1_42_ and r*Py*MSP8, was not due to an improved conformation of protective B cell epitopes or the generation of novel epitopes in the chimeric molecule [19, 25]. The key finding was that immunization with r*Py*MSP1/8 elicited an MSP8-restricted T cell response that provided effective help for both *Py*MSP1_19_ and *Py*MSP8 specific B cells to produce high and sustained levels of protective antibodies [25].

Based on the proof-of-concept studies in the *P. yoelii* model, *P. falciparum* MSP8 was pursued as a parasite-specific carrier protein to overcome challenges associated with the production of recombinant antigen vaccines (quality, yield) and with the sub-optimal immunogenicity of relevant neutralizing B cell epitopes [26, 27]. Among different *P. falciparum* isolates, MSP8 is highly conserved, exhibiting 95 % amino acid identity with slight variations in an N-terminal Asn/Asp-rich domain [28]. The remaining C-terminal sequence is invariant. Following codon harmonization [29] and genetic fusion of *Pf*MSP1_19_ and *Pf*MSP8 (ΔAsn/Asp) coding sequences, a properly folded chimeric antigen was produced in high yield, utilizing an *Escherichia coli* expression system [27]. Immunogenicity studies in both inbred and outbred mice demonstrated a strong T cell response restricted to epitopes within *Pf*MSP8 sequence and the production of high titres of antibodies to both MSP1_19_ and MSP8 domains. Comparable *Pf*MSP1/8 immunogenicity studies in rabbits demonstrated the induction of high titres of *Pf*MSP1_19_ specific antibodies that very effectively inhibited the in vitro growth of *P. falciparum* parasites of both the 3D7 and FVO strains. While these data are encouraging, results of vaccine studies in mice and rabbits do not always predict outcomes upon immunization of human subjects. In this study, the immunogenicity of *Pf*MSP1/8 was evaluated in non-human primates, assessing the magnitude, specificity and functional activity of elicited antibodies.

## Methods

### Recombinant antigens

The production and purification of the chimeric r*Pf*MSP1/8 and r*Pf*MSP8 (ΔAsn/Asp) (*P. falciparum* FVO strain) followed the same protocol, using codon-harmonized, synthetic gene sequences cloned into pET-28 (EMD Biosciences, San Diego, CA, USA) and SHuffle™ T7 Express *lysYE. coli* cells (New England Biolabs, Ipswich, MA, USA) as host. Expression of the recombinant proteins was accomplished using a BioFLo115 bench-top bioreactor (New Brunswick Scientific, Edison, NJ, USA). Protocols for the expression and purification of recombinant antigens have been reported previously [26, 27]. For this study, r*Pf*MSP1/8 and r*Pf*MSP8 (ΔAsn/Asp) were further purified by gel filtration (Superdex 75, GE Healthcare Bio-Sciences Corp, Piscataway, NJ, USA) followed by a second round of Ni–NTA affinity chromatography (Qiagen, Valencia, CA, USA). The eluted material was dialyzed into 20 mM Tris–HCl, pH 7.2, 0.5 M NaCl. The final preparations of r*Pf*MSP1/8 and r*Pf*MSP8 (ΔAsn/Asp) were filter sterilized (0.22 µm), aliquoted and stored at −80 °C. The final protein concentration was determined by bicinchoninic acid protein assay (BCA; Thermo Scientific, Rockford, IL, USA). Endotoxin levels were quantitated using the ToxinSensor Chromogenic LAL Endotoxin Assay (GenScript, Piscataway, NJ, USA). Protein purity and conformation were assessed by Coomassie blue staining following SDS-PAGE on 10 % gels, run under both reduced and non-reduced conditions. Purity (reduced lanes) and  % monomer (doublet, non-reduced lanes) were estimated by densitometry using ImageJ processing and analysis software [30].

### Animals and immunizations

Adult *Aotus nancymaae* monkeys were housed at a Centers for Disease Control (CDC) primate facility, fully-accredited by the Association for Assessment and Accreditation of Laboratory Animal Care International (AAALAC). Animal studies were reviewed, approved and conducted in compliance with the Institutional Animal Care and Use Committee (IACUC) of CDC. Eighteen *Aotus* monkeys were stratified according to weight and sex into three groups of six animals, which were then randomly assigned to vaccine and control groups. On day 0, groups of animals were immunized by intramuscular injection (0.5 ml) of 50 µg of r*Pf*MSP1/8 or r*Pf*MSP8 (ΔAsn/Asp) emulsified in Montanide ISA 720 (Seppic Inc, Paris, France) at a ratio of 70:30 (vol/vol). Control animals were immunized with saline/Montanide alone. Monkeys were boosted on day 28 and on day 84 with the same antigen/adjuvant formulation that was used for the priming immunization.

### Safety assessment

All monkeys were in good health, free of tuberculosis and weighed between 0.8 and 1.2 kg at the start of the study. Animals were weighed at weekly intervals. Blood and serum were collected at biweekly intervals beginning 2 weeks prior to the first immunization and until the completion of the study. Immunization sites were monitored continuously for adverse local reactions. Systemic reactions were evaluated by monitoring a panel of haematologic parameters and serum chemistry values that included measurements of white blood cells, red blood cells, haemoglobin, haematocrit, platelets, blood urea nitrogen, glucose, alkaline phosphatase, total protein, alanine aminotransferase, and creatinine.

### Measurement of antigen-specific antibody responses by ELISA

Antigen-specific antibody responses induced by immunization with r*Pf*MSP1/8 and r*Pf*MSP8 (ΔAsn/Asp) were measured by ELISA as previously described [27]. Plates were coated with 0.25 µg per well of purified r*Pf*MSP1/8, r*Pf*MSP8 (ΔAsn/Asp), rGST-*Pf*MSP_19_ (FVO) or rGST-*Pf*MSP_19_ (3D7). Pre-immune sera and sera collected 2 weeks following primary, secondary and tertiary immunizations were serially diluted twofold starting at 1:1250. Bound antibodies were detected using horseradish peroxidase conjugated goat anti-*Aotus* IgG [31] diluted 1:7500 and with ABTS [2,2′-azinobis(3-ethylbenzthiazolinesulfonic acid)] as substrate. *A*_405_ values between 1.0 and 0.1 were plotted and titre calculated as the reciprocal of the dilution that yielded an *A*_405_ of 0.5. A high titre pool of serum obtained from r*Pf*MSP1/8 immunized monkeys (n = 5) was included on each assay plate as an internal reference to normalize the data between assays. Pre-immune sera were used to establish background reactivity of each animal to each antigen and subtracted as background.

### *Plasmodium falciparum* growth inhibition assays

The growth inhibitory activity (GIA) of protein G purified IgG from r*Pf*MSP1/8 to r*Pf*MSP8 (ΔAsn/Asp) immunized and adjuvant control monkeys was assessed in vitro by the measurement of parasite lactate dehydrogenase activity [32] using standard protocols. Pre-bleed and adjuvant control IgG served as negative controls. Immune IgG was tested at a concentration of 10 mg/ml. GIA was calculated relative to *P. falciparum* FVO blood-stage parasites growing in complete media in the absence of any added *Aotus* IgG.

### Statistical analysis

When comparing data from two groups, the statistical significance of the differences in antigen-specific IgG titres or parasite growth inhibition was determined by the Mann–Whitney test. The statistical significance of increases in antigen-specific titres between paired primary, secondary and tertiary immunization sera was determined using the Friedman test followed by Dunn’s multiple comparison correction. Probability (*p*) values ≤ 0.05 were considered significant.

## Results

**Fig. 1:**
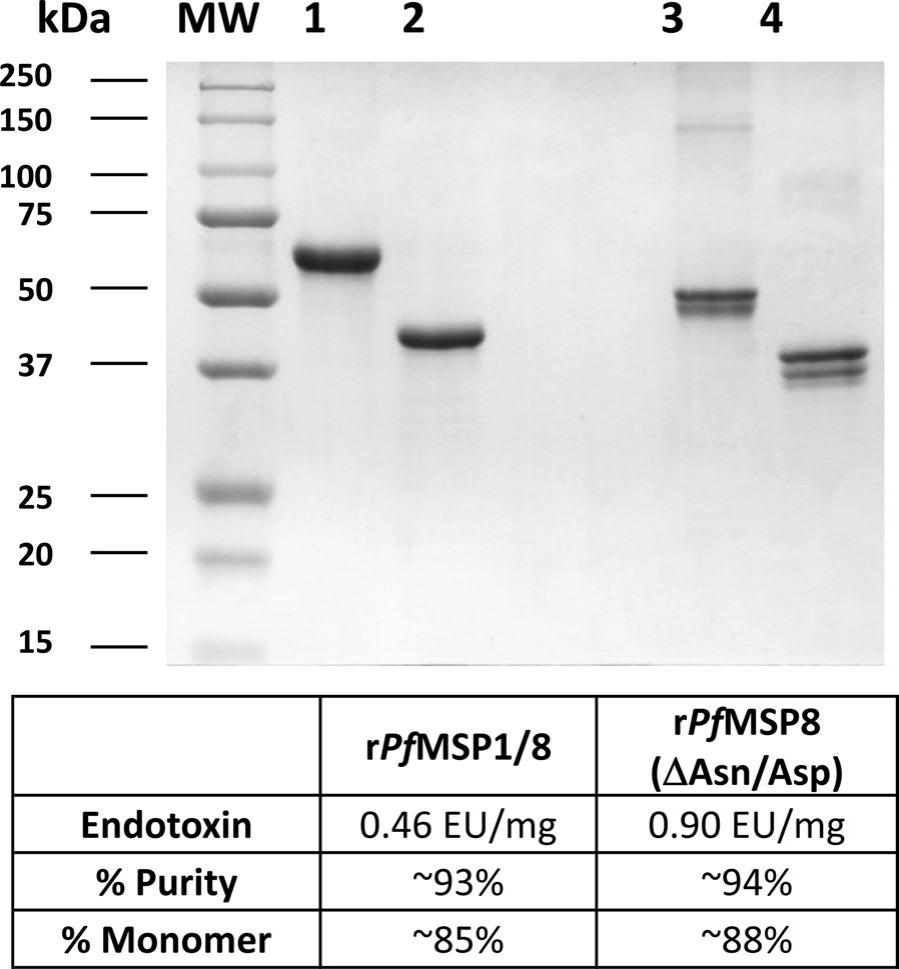
Purified recombinant chimeric r*Pf*MSP1/8 and r*Pf*MSP8 (ΔAsn/Asp) vaccine antigens. A Coomassie blue-stained 10 % SDS–polyacrylamide gel containing purified r*Pf*MSP1/8 (3 μg, *lanes* 1 and 3) or r*Pf*MSP8 (ΔAsn/Asp) (3 μg, *lanes 2* and *4*) was run under reducing (*lanes q* and *2*) and non-reducing (*lanes 3* and *4*) conditions. Molecular weight markers in kilodaltons (kDa) are shown. Endotoxin levels and  % purity for each antigen preparation are also indicated

The production and purification of r*Pf*MSP1/8 and r*Pf*MSP8 (ΔAsn/Asp) was reported previously [26, 27]. Figure 1 shows a Coomassie-blue stained polyacrylamide gel of reduced (lanes 1 and 2) and non-reduced (lanes 3 and 4) purified recombinant antigens used in this *Aotus* immunogenicity study. As previously observed, the two recombinant vaccine antigens migrate as a predominant doublet in the absence of reducing agent [26, 27]. Purity of the final product was estimated to be 93–94 % with ≥85 % monomer. Endotoxin levels in the final vaccine preparation were less than 1 EU/mg of protein. Prior studies showed that immunization of *Aotus* monkeys with r*Pf*MSP1_42_ elicited antibodies that inhibited the in vitro growth of *P. falciparum* blood-stage parasites [31]. r*Pf*MSP1_42_ vaccines formulated with Complete Freund’s adjuvant elicited higher titres of functional antibodies in comparison to formulations with Montanide ISA 720. For this study, Montanide ISA 720 was selected as adjuvant in an effort to balance potency with safety. *Aotus* monkeys (six per group) were immunized and boosted twice (days 0, 28 and 84) with 50 µg/dose of r*Pf*MSP1/8 or r*Pf*MSP8 (ΔAsn/Asp) formulated with Montanide ISA 720 or with adjuvant alone.

Immunization with r*Pf*MSP1/8 or r*Pf*MSP8 (ΔAsn/Asp) was reasonably well tolerated. Local injection site reactions were noted following the second and third immunizations (Table 1). These consisted primarily of small nodules at the injection site and/or local swelling, which typically resolved. Prior to the first immunization, baseline haematology and blood chemistry profiles were determined for each animal. Subsequently, blood and serum were collected biweekly through the course of the study for monitoring. Fluctuations from baseline values were noted in individual animals across groups. Alterations attributable to a systemic response to a specific vaccine formulation were not apparent.

**Table 1:** Immunization site reactions

Group I Montanide ISA 720 control	
AI-1944	Small bump above inoculation site, right side following first immunization, persisting
AI-2010	None
AI-2016	Haemorrhage at venipuncture site, fatal
AI-2029	None
AI-2033	Hard knot, right side following third immunization
AI-2036	None
Group II r*Pf*MSP8 (ΔAsn/Asp) + Montanide ISA 720	
AI-1990	None
AI-1993	None
AI-2012	Right thigh slightly swollen, one time point following second immunization
AI-2022	Right thigh slightly swollen, one time point following second immunization
AI-2028	Swollen dime-sized knot, reduced at subsequent time point
T-1048	None
Group III r*Pf*MSP1/8 + Montanide ISA 720	
AI-1924	Right thigh larger, no focus, one time point following second immunization
AI-2006	Left side, large knot, following second immunization, persisting Right side, swollen, hot dime-size; reduced by next time point
AI-2024	None
AI-2030	None
AI-2031	Right thigh swollen, resolved following second immunization
AI-2035	Right thigh swollen, one time point following second immunization

**Fig. 2:**
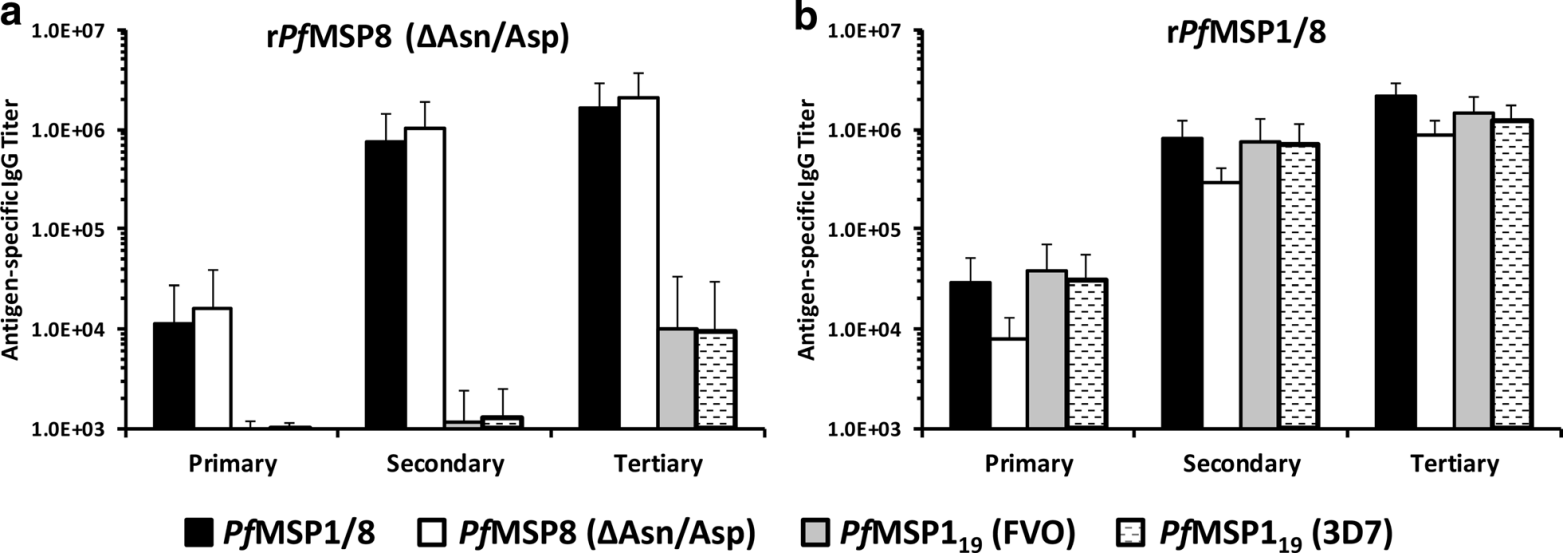
Specificity of antibody response induced by immunization with r*Pf*MSP8 (ΔAsn/Asp) versus r*Pf*MSP1/8 vaccines. Antigen-specific IgG titres (mean ± standard deviation) in sera collected from *Aotus* monkeys (6 animals/group) immunized with **a** r*Pf*MSP8 (ΔAsn/Asp) or **b** r*Pf*MSP1/8 vaccines formulated with Montanide ISA 720 were determined by ELISA. Sera collected 2 weeks following primary, secondary and tertiary immunization were evaluated. ELISA plates were coated with either r*Pf*MSP1/8, r*Pf*MSP8 (ΔAsn/Asp), rGST-*Pf*MSP1_19_ (FVO), or rGST-*Pf*MSP1_19_ (3D7) as indicated. For each animal, reactivity of pre-immunization serum was subtracted as background

Sera collected 2 weeks after each immunization were evaluated by ELISA to determine the titres of IgG antibodies specific for the r*Pf*MSP8 (ΔAsn/Asp) carrier and for *Pf*MSP1_19_ epitopes of both the FVO and 3D7 alleles (Fig. 2). Immunization with r*Pf*MSP8 (ΔAsn/Asp) or r*Pf*MSP1/8 elicited comparable IgG responses to r*Pf*MSP8 specific epitopes following the primary immunization (*p* > 0.5). Two additional immunizations markedly boosted the r*Pf*MSP8-specific IgG response in both groups by >100-fold (*p* < 0.05, primary vs tertiary mean titre). Significant titres of antibodies to *Pf*MSP1_19_ epitopes were not detected in r*Pf*MSP8 (ΔAsn/Asp) immunized animals following primary and secondary immunization. Following a third immunization with the r*Pf*MSP8 (ΔAsn/Asp) carrier, a low titre of antibodies cross-reactive with *Pf*MSP1_19_ was detected in two animals, presumably due to some similarity in the C-terminal EGF-like domains of *Pf*MSP1 and *Pf*MSP8 [33]. In contrast, immunization with r*Pf*MSP1/8 induced a strong and boost-able antibody response to *Pf*MSP1_19_ that was highly cross-reactive between the FVO and 3D7 alleles of *Pf*MSP1_19_.

**Fig. 3:**
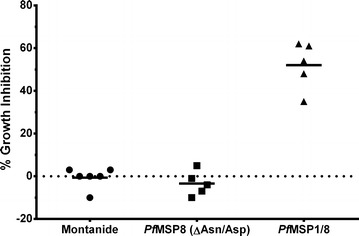
In vitro inhibition of *Plasmodium falciparum* growth by *Aotus* IgG induced by immunization with r*Pf*MSP8 (ΔAsn/Asp) versus r*Pf*MSP1/8 vaccines. In vitro growth inhibitory activity of IgG from immunized *Aotus* monkeys for *P. falciparum* FVO blood-stage parasites was based on measurement of parasite lactate dehydrogenase levels. Purified IgG (10 mg/ml) from animals immunized three times with Montanide alone (n = 6), r*Pf*MSP8 (ΔAsn/Asp) + Montanide (n = 5) or r*Pf*MSP1/8 + Montanide (n = 5) was evaluated. Two immunized animals (1 r*Pf*MSP8 (ΔAsn/Asp), 1 r*Pf*MSP1/8) tested at less than 10 mg/ml of IgG were not included in the Figure. Per cent growth inhibition in the presence of *Aotus* IgG relative to controls in the absence of IgG is shown

To assess functionality, antibodies elicited by immunization with r*Pf*MSP8 (ΔAsn/Asp) or r*Pf*MSP1/8 were evaluated for the ability to inhibit the in vitro growth of *P. falciparum* blood-stage parasites. As shown in Fig. 3, purified IgGs obtained following the third immunization of *Aotus* monkeys with r*Pf*MSP1/8 effectively inhibited parasite growth. In contrast, no growth inhibitory activity was observed with IgG from r*Pf*MSP8 (ΔAsn/Asp) immunized or adjuvant control animals (*p* < 0.01). *Pf*MSP8 is not believed to be a significant target of growth inhibitory antibodies due to its low and transient expression on the merozoite surface [26, 34]. These data from *Aotus* monkeys are also consistent with the previous findings in antigen reversal assays, demonstrating that the inhibitory activity of rabbit anti-r*Pf*MSP1/8 IgG was due to antibodies specific for *Pf*MSP1_19_ epitopes [27]. However, there was no correlation between GIA and the magnitude of the antibody response to r*Pf*MSP1/8, r*Pf*MSP8 (ΔAsn/Asp) or r*Pf*MSP1_19_ in r*Pf*MSP1/8 immunized animals. As such, the fine specificity of the *Pf*MSP1_19_ response and/or a potential interaction between *Pf*MSP1_19_ and *Pf*MSP8 specific antibodies may be a factor.

Following immunization, r*Pf*MSP8 (ΔAsn/Asp), r*Pf*MSP1/8 and adjuvant control groups were challenged intravenously with 50,000 *P. falciparum* FVO parasitized red blood cells (pRBCs). Three r*Pf*MSP8 (ΔAsn/Asp) immunized animals developed moderate parasitaemia (<200,000 pRBC/µl) and self-cured. The remaining three animals in this group developed high-density parasitaemia (>200,000 pRBC/µl) requiring drug treatment to clear the infection. Similarly, three r*Pf*MSP1/8 immunized animals controlled parasitaemia (<200,000 pRBC/µl), with two of these clearing their infection and one requiring drug treatment due to anaemia. In the Montanide control group, three animals developed high-density parasitaemia (>200,000 pRBC/µl) and were drug treated to clear the infection. However, the remaining two adjuvant control animals exhibited greatly extended prepatent periods, developed unexpectedly low peak parasitaemia (<5000 pRBC/µl) and rapidly self-cured. The reason for this large and unusual variability in parasitaemia in the control group with this highly virulent strain was not apparent. This line of *P. falciparum* FVO strain in almost every previous instance has produced rapidly rising parasitaemia in control animals that required drug treatment to prevent death. Nevertheless, because of this unexpected variability, the ability to draw meaningful conclusions regarding vaccine efficacy was compromised.

In a previous study, Lyon et al. [31] completed an immunogenicity study in *Aotus* monkeys with r*Pf*MSP1_42_ formulated with CFA/IFA, AS02A or Montanide 720. With the exception of adjuvant choice, immunization protocols were similar to the present study with respect to immunizing dose, route, number of immunizations, and interval between immunizations. However, antibodies elicited by immunization with *Pf*MSP1_42_ formulated with AS02A or Montanide 720 only modestly inhibited *P. falciparum* growth. The induction of more effective inhibitory antibodies required immunization with *Pf*MSP1_42_ formulated with Freund’s adjuvant. In contrast, IgG elicited following immunization with the *Pf*MSP1/8 chimeric vaccine formulated with Montanide ISA 720, an adjuvant which has been used in human clinical trials, potently inhibited parasite growth (52.0 ± 11.3 %).

These data, in conjunction with prior studies [26, 27], demonstrate that r*Pf*MSP8 (ΔAsn/Asp) is an effective malaria-specific carrier protein that elicits strong CD4+ T cell help for the production of merozoite neutralizing antibodies to linked *Pf*MSP1_19_ epitopes. The potency of r*Pf*MSP8 (ΔAsn/Asp) has been demonstrated in mice and rabbits and now in non-human primates and does not require Freund’s adjuvant. Upon immunization of human subjects, similarly strong CD4+ T cell responses to provide B cell help are expected as r*Pf*MSP8 (ΔAsn/Asp) is predicted to contain epitopes that will bind with high affinity to multiple HLA class II alleles [35]. It is also notable that r*Pf*MSP8 (ΔAsn/Asp) has been engineered to facilitate production and purification of chimeric antigens at high levels and in native conformation utilizing readily scalable protocols and procedures. By fusing relevant neutralizing B cell epitopes of other vaccine candidates to *Pf*MSP8 (ΔAsn/Asp), a conserved, highly immunogenic *P. falciparum*-specific carrier protein, it may be possible to similarly to overcome challenges associated with the production of recombinant antigen vaccines (quality, yield) and sub-optimal immunogenicity.

## Conclusions

Extending previous work in small animal models, these studies now show that the chimeric *Pf*MSP1/8 vaccine is highly immunogenic in non-human primates and induces potent parasite neutralizing antibodies. These data justify additional effort to incorporate *Pf*MSP1/8 as a component of a multivalent *P. falciparum* malaria vaccine to augment control efforts. r*Pf*MSP8 (ΔAsn/Asp) may have broader use as a fusion partner for poorly immunogenic and/or difficult to produce sub-unit vaccine candidates.
